# Suppression of Autoimmune Arthritis by Small Molecule Inhibitors of the JAK/STAT Pathway

**DOI:** 10.3390/ph3051446

**Published:** 2010-05-12

**Authors:** Charles J. Malemud

**Affiliations:** Division of Rheumatic Diseases, Departments of Medicine & Anatomy, School of Medicine, Case Western Reserve University, Cleveland, Ohio 44106, USA; E-Mail: cjm4@cwru.edu; Tel: +1-216-844-7846; Fax: +1-216-844-2288

**Keywords:** arthritis, autoimmune, Janus kinase, signal transducers and activators of transcription, small molecule inhibitor

## Abstract

A skewed ratio of pro-inflammatory to anti-inflammatory cytokines, elevated growth factor synthesis and T- and B-lymphocyte activation are 3 hallmarks of rheumatoid arthritis (RA) pathology. Interleukin-6 (IL-6), IL-7, IL-17, IL-12/IL-23 and growth factors, granulocyte macrophage-colony stimulating factor, IL-3, and erythropoietin activate the Janus Kinase/Signal Transducers and Activators of Transcription (JAK/STAT) pathway. Evidence showed that STAT protein phosphorylation (p-STAT) by activated JAKs is permissive for p-STAT to act as transcription factors by binding to STAT-responsive gene promoter sequences. This event is critical for perpetuating RA, in part, by up-regulating pro-inflammatory cytokine gene transcription. Activation of JAK/STAT by cytokines and growth factors can induce ‘cross-talk’ with other signaling pathways by which Stress-Activated Protein/Mitogen-Activated Protein Kinase (SAP/MAPK) and Phosphatidylinositide-3-Kinase (PI3K)-mediated signaling are also activated. JAK-specific small molecule inhibitors (SMIs) were developed to test whether JAK/STAT pathway blockade would regulate autoimmune-mediated inflammation. JAK-specific SMI blockade inhibited p-STAT induced by pro-inflammatory cytokines *in vitro*. Systemically administered JAK-specific SMI blockade also ameliorated biomarkers of inflammation in well-validated arthritis animal models. A few JAK-specific SMIs have made their way into RA clinical trials. In fact, the JAK3-specific SMI, CP-690,500 is the first JAK/STAT SMI to be assessed for clinical efficacy in a Phase III RA trial.

## 1. Introduction

Rheumatoid arthritis (RA) is a systemic autoimmune disorder of unknown etiology characterized by chronic inflammation in multiple synovial joints and other organs [1]. The inflammatory response in synovial joint tissue is initiated by defective innate and acquired immunity which principally involves heightened Toll-like and ‘nucleotide-binding domain leucine-rich repeat containing’ protein NLR/NOD-like receptor activity [2], dysfunctional T-helper cell (*i.e.* CD4^+^) responses, [3] mature B-cell hyperactivity resulting in autoantibody production [4,5], aberrant antigen presenting cell (APC) activity including markedly elevated responses to antigen driven by activated dendritic cells (DCs) [6] and an overall loss of immune tolerance exemplified by inadequate T-regulatory (T_reg_) cell functional responses [7]. The autoimmune-mediated inflammatory response in RA is also characterized by an exuberant recruitment, and retention of macrophages, and mast cells within the synovial lining tissue. Of note, in RA joints the vast majority of neutrophils are found in the synovial fluid rather than in the synovial lining layer. Recruitment and retention of inflammatory cells is driven by elevated levels of chemokines and adhesion molecules which results in synovial tissue hyperplasia with pannus development [8]. 

A skewing of the cytokine repertoire produced by the T_h_1 and T_h_2 T-cell subsets mainly causes the over-production of pro-inflammatory cytokines exemplified by tumor necrosis factor-α (TNF-α), interleukin-1 (IL-1), IL-2, IL-6, IL-7, IL-12/IL-23 and IL-17 at the expense of anti-inflammatory cytokine production [9]. Pro-inflammatory cytokine gene up-regulation is also typified by elevated levels of IL-2, IL-3, IL-13, granulocyte/monocyte-colony stimulating factor (GM-CSF), leukemia inhibitory factor and Type I cytokine receptor activation or in the case of interferon-α, β, γ interactions with either Type I or Type II receptors. Engagement of either Type I or Type II cytokine receptor causes activation of the Janus Kinase/Signal Transducers and Activators of Transcription (JAK/STAT) signaling pathway [10,11,12]. The principal consequence of JAK activation is STAT protein phosphorylation (*i.e.*, p-STAT) which converts activated STAT protein dimers to fully functional and potent transcription factors that can bind to STAT-target genes many of which regulate the transcription of pro-inflammatory cytokine genes [12,13,14,15,16,17]. 

In addition, in adult RA pro-inflammatory cytokine gene up-regulation can also occur directly via activation of the Stress-Activated Protein/Mitogen-Activated Protein Kinase (SAP/MAPK) pathway or by ‘cross-talk’ after JAK/STAT activation [12]. Activation of the SAP/MAPK pathway causes excessive levels of matrix metalloproteinases (MMPs) [18,19] and the disintegrin and metalloproteinase (ADAMS) enzyme family [20] to be synthesized resulting in the degradation of articular cartilage extracellular matrix proteins [18,19] and stimulation of vascular invasion into synovial tissue by neoangiogenesis [20]. Furthermore, the unregulated development of osteoclasts from osteoclastic-progenitor monocyte precursors under the influence of osteoclastogenesis-stimulating proteins NFATc1 [21] and RANKL [22,23] is ultimately responsible for the irreversible destruction of subchondral bone via proteinase-mediated degradation. Finally, hyperactivation of nuclear factor-κB (NF-κB) and over stimulation of the PI3K/Akt/Protein Kinase B/mammalian target of rapamycin (PI3K/Akt/PKB/mTOR) ‘cell survival’ pathway [24,25,26,27] are also likely to be responsible for the high level of apoptosis-resistance that is typically a characteristic of inflamed RA synovial tissue. The reader is referred to a recently published schematic diagram that defined the ‘cross-talk’ relationships between cytokine and growth factor-induced activation of the JAK/STAT, SAP/MAPK and PI3K/Akt pathways [12].

The critical role played by pro-inflammatory cytokines as deregulators of T-cell proliferation, B-cell hyperactivity and chemokines and adhesion molecule production led to the development of several anti-rheumatic biological agents [reviewed in 8] which have proven to show efficacy in the clinical therapy of adult RA. However, since activation of the JAK/STAT pathway has also been recognized to be highly involved in perpetuating the progression of adult RA pathology, the construction of small molecule inhibitors (SMIs) that specifically block activation of JAK/STAT has been the focus of several *in vitro* studies using tumor cells [28,29,30,31,32,33,34,35] and as a possible therapy for kidney transplant rejection [36]. An assessment of their effectiveness in well-validated animal models of RA has also proven to be informative [12]. For example, The JAK3-specific SMI, CP-690,550 was first assessed in 2 well-validated rodent models of RA where reduced inflammatory cell influx, joint damage and preservation of cartilage structure in the presence of the drug was demonstrated [37]. CP690,550 is the first JAK3-specific SMI to show safety and efficacy in a Phase IIa RA clinical trial [38]. Presently, CP-690,550 is being evaluated for its efficacy in a larger Phase III RA clinical trial. Additional JAK-specific SMIs are under development for use as future cancer and RA therapies as discussed below. 

## 2. Novel JAK-specific SMIs: Evaluation on Tumor Cells and Potential Role in RA Therapy

### 2.1. Nb-(alpha-hydroxynaphthoyl)serotonin (MS-1020)

MS-1020 is a novel JAK3 inhibitor [39]. MS-1020 was shown to effectively block constitutively active JAK3 in the Hodgkin lymphoma cell lines, L540 and HLDM-2 and in the MDA-MB-468 breast cancer cell line [38]. In addition, MS-1020 suppressed IL-2-induced JAK3/STAT5 activation but not prolactin-stimulated JAK2/STAT5 signaling in rat T-lymphocyte Nb2 cells. Further studies demonstrated that MS-1020 blocked JAK3 activity through its ability to bind to the JAK3 catalytic site. Importantly, MS-1020 induced apoptosis and decreased cell survival by down-regulating the anti-apoptosis genes, Bcl-2, Bcl-xL, Mcl-1 and survivin. 

While there is a relative paucity of data regarding the exact nature and role of p-STAT5, compared to, for example, the role of p-STAT3 in RA the results of 3 recent studies lends support to a role for p-STAT5 in RA which can be summarized as follows: (1) RA synovial fibroblasts synthesized only low levels of constitutive CCL13/monocyte chemotractant protein-4. However, oncostatin M increased CCL13 production via activation of STAT5, ERK 1/2 and p38 kinase [40]; (2) Elevated levels of granzyme B were produced by human plasmacytoid DCs which was regulated at the transcriptional level by JAK1, STAT3, STAT5 and IL-3, a cytokine produced by activated T-cells [41]; and (3) in a study to improve the antibody selectivity for neutralizing the biological activity of GM-CSF, Steidl *et al.* [42] found that the neutralizing potential of antibody MOR 04357 on human premyeloid cell line TF-1 proliferation was accomplished via blockade of the GM-CSF receptor by MOR 04357 as well as abolition of p-STAT5. Thus in keeping with results of the studies cited above, the significance of MS-1020 blockade of JAK3/STAT5 activation may be relevant to the role of STAT5 in RA. In that regard, Zhou *et al*. [43] had previously shown that CD4^+^ T-cells when capable of expressing CD80 (a DC plasma membrane surface marker), showed a sustained level of proliferation even in the absence of APCs. Furthermore, T cells expressing CD80 continued to produce activated NF-κB and activator protein-1 for 24 h after separation from APCs. Of note, CD4^+^ T-cells expressing CD80 also showed evidence of up-regulated STAT5 in the absence of APCs or exogenous signal-1. These results suggested that STAT5 activation could be important in sustaining CD4^+ ^ T-cell proliferation in the absence of APCs *in vitro* but only when CD4^+ ^ T-cells had acquired the capacity to express CD80. Finally, Baldwin *et al*. [44] recently showed that DCs derived from RA patients after therapy with either the soluble TNF receptor (TNFR) p55 Ig fusion protein, etanercept or the chimeric anti-TNFα monoclonal antibody infliximab showed impaired up-regulation of CD80 and CD86 after stimulation with lipopolysaccharide *in vitro* as well as poor T-cell stimulatory activity.

### 2.2. 2-[(3,5-bis-Trifluoromethylphenyl)-hydroxymethyl]-1-(4-nitrophenylamino)-6-phenyl-1,2,4a,7a-tetrahydropyrrolo[3,4-b]-pyridine-5,7-dione (AUH-6-96)

The novel JAK/STAT SMI, AUH-6-96 reduced p-STAT3 and its downstream target, SOCS3 in Hodgkin lymphoma L540 cells by blocking constitutively active JAK3 [45]. Moreover, AU-6-96 only blocked JAK3 activation in cell lines showing aberrant JAK/STAT signaling. AU-6-96 also induced apoptosis in L540 cells by down-regulating the p-STAT3-regulated downstream anti-apoptosis protein, Bcl-xL. However, a note of caution regarding the interpretation of these results appears to be appropriate. Thus, although Kim *et al.* [45] showed that AUH-6-96 inhibited p-STAT3 and JAK3 but not p-STAT2, this study failed to screen AUH-6-96 for its activity against JAK1, the other critical activator of STAT3. In summary, AU-6-96 may ultimately prove to be useful for regulating the uncontrolled proliferation of cultured RA-synovial fibroblasts where defective apoptosis has been demonstrated [25,46].

### 2.3. INCB018424

A novel pyrazol-3-ylamino pyrazine JAK2 inhibitor, INCB018424 [47,48] was shown to inhibit IL-6-induced proliferation of the JAK2V^617^F-positive Ba/F3 cancer cell line [49]. Furthermore, orally-administered INCB018424 suppressed splenomegaly and circulating cytokines in a mouse model of JAK2V^617^F-induced myeloproliferative disease by effectively eliminating neoplastic cells which was accompanied by increased survival of the mice but without inducing myelosuppression or immunosuppression. In a recent 28-day RA clinical trial involving 12 patients who were orally-administered INCB018424, 83% achieved an American College of Rheumatology 20 (ACR20) criteria compared to 75% in the placebo arm, whereas 58% achieved an ACR50 response (0% placebo) and 33% an ACR70 response (0% placebo) [50]. Of note, INCB018424 also inhibited IL-6-induced p-STAT3 in whole blood cell cultures derived from both RA and normal subjects. In addition, orally-administered INCB018424 suppressed IL-6 and CD40 levels measured in the plasma of RA patients. 

Effective suppression of RA clinical symptoms was already achieved with tocilizumab, an anti-IL-6-receptor (IL-6R) monoclonal antibody [8,12,17]. The evidence for the clinical efficacy of tocilizumab in the therapy of RA suggested that effective suppression of IL-6-induced JAK/STAT activation was likely the operative mechanism responsible for its clinical effectiveness [17]. However, evidence from several RA clinical trials involving tocilizumab treatment of RA patients indicated that biomarkers of liver function will require constant monitoring over time [12,17]. This suggested that alternative strategies to neutralizing IL-6-mediated *trans*-signaling responses by inhibition of IL-6/IL-6R binding must also be considered [51]. Thus, the initial evidence for efficacy of INCB018424 in RA clinical trials warrants further analysis. 

### 2.4. N-tert-Butyl-3-(5-methyl-2-(4-(4-methylpiperazin-1-yl)phenylamino)pyrimidin-4-ylamino)-benzenesulfonamide (TG101209)

TG101209 is a novel SMI with a strong and relatively selective inhibitory effect on JAK2 (IC_50_, 6nM) *vs.* JAK3 (IC_50_, 169nM); Fms-related tyrosine 3 (Flt3) (IC_50_, 25nM) and ‘rearranged-during-transfection’ (RET) (IC_50_, 17nM) kinases [52]. TG101209 effectively inhibited the growth of Ba/F3 cells expressing the JAK2V^617^F or MPLW^515^L/K mutations (IC_50_, ~200nM) [52]. Additionally, TG101209 induced cell cycle arrest and apoptosis in JAK2V^617^F-expressing acute myeloid leukemia cells which was accompanied by inhibition of p-JAK2V^617^F, p-STAT5 and p-STAT3. TG101209 also showed therapeutic efficacy in nude mice. More recently, Wang *et al*. [53] showed that TG101209 in combination with panobinostat (LBH589), a histone deacetylase inhibitor [54,55], demonstrated greater cytotoxicity towards primary CD34^+ ^ cells derived from subjects with BCR-ABL-negative myeloproliferative disease than CD3^+^ cells from normal donors. Thus, TG101209 may be amenable for testing of its capacity to induce apoptosis specifically on RA synovial tissue. Table 1 summarizes the major findings of each of these experimental studies. 

**Table 1:** JAK-Selective Small Molecule Inhibitors Suppress Proliferation and Induce Apoptosis in Cancer Cell Lines.

*SMI*	*JAK/STAT Selectivity*	*Response*	*Reference*
MS-1020	JAK3	↑ Apoptosis^1^	[39]
		↓ Bcl-2, Bcl-xL,	
		Mcl-1, survivin	
AUH-6-96	p-STAT3	↑ Apoptosis^2^	[45]
		↓ Bcl-xL	
INCB018424	JAK2	↓ IL-6-induced	[49]
		Proliferation^3^	
TG101209	JAK2	↓ Proliferation^4^	[52]
		↑ Apoptosis	

^1^ L540 and HLDM-2 Hodgkin lymphoma cells; MDA-MB-468 breast cancer cells. ^2^ L540 cells. ^3^ Ba/F3 cells with the JAK2V^617^F mutation. ^4^ Ba/F3 cells with the JAK2V^617^F or MPLW^515^L/K mutation.

## 3. Conclusions

There is now compelling evidence from cell culture and animal studies that excessive levels of pro-inflammatory cytokines produced by RA monocytes and T-cells best exemplified by IL-2, IL-6, IL-12/IL-23 and IL-17 are potent activators of the JAK/STAT pathway. Deregulated JAK/STAT activation is now accepted as playing a critical role in perpetuating RA pathology through its capacity to produce p-STAT proteins which acting as transcription factors up-regulate pro-inflammatory cytokine gene activity. Of note, JAK/STAT pathway activation can also result in the activation of the SAP/MAPK pathway which is, in part, responsible for MMP gene up-regulation and also in the activation of the PI3K/Akt/mTor pathway which appears to be the critical driver of anti-apoptosis responses in RA inflamed synovial tissue [56]. The results of recent studies using JAK-selective SMIs in cancer cell lines have demonstrated their capacity to suppress proliferation to induce apoptosis 

In clinical trials involving active RA patients, the initial evidence indicated that JAK/STAT SMIs appeared to be more consistently effective than SAP/MAPK SMIs [57,58], especially those developed to inhibit specific p38 kinase isoforms [59,60,61] for suppressing disease activity. In fact, CP-690,550, a JAK3-specific SMI has now entered a Phase III RA clinical trial with the results forthcoming. Finally, a group of novel JAK-selective SMIs have now been tested on various tumor cell lines with evidence for their capacity to suppress cell proliferation and induce apoptosis thus making them excellent candidates for testing in cultures of RA synovial fibroblasts and in well-validated animal models of RA. 
